# Effects of dietary medium-chain fatty acid supplementation levels on growth performance, blood parameters, fecal score and metabolites in nursery pigs

**DOI:** 10.5713/ab.25.0099

**Published:** 2025-05-19

**Authors:** Chan Ho Kwon, Jannell A. Torres, Madison J. Mejia, Eva S. Safaie, Joseph G. Usack, Young Dal Jang

**Affiliations:** 1 Department of Animal and Dairy Science, University of Georgia, Athens, GA, USA; 2 Department of Food Science and Technology, University of Georgia, Athens, GA, USA

**Keywords:** Antioxidant Status, Fecal Score, Free Fatty Acids, Growth Performance, Medium-chain Fatty Acids, Nursery Pigs

## Abstract

**Objective:**

This study evaluated the effects of increasing medium-chain fatty acid (MCFA) supplementation levels on growth performance, blood parameters, fecal score, and metabolites in nursery pigs.

**Methods:**

A total of 100 newly weaned pigs (initial body weight: 6.68±0.97 kg) were allotted to 5 treatments in 5 replicates, with 4 pigs per pen for a 28-d feeding trial. Treatments were MCFA supplementation levels at 0.0%, 0.4%, 0.8%, 1.2%, and 1.6%. The 2% soybean oil in the basal diet was replaced with MCFA (a 50:50 blend of free caproic and caprylic acids) on a weight-to-weight basis.

**Results:**

Increasing MCFA levels tended to linearly increase the final body weight (p = 0.06) and average daily gain in the overall period (p = 0.06), with no difference in overall average daily feed intake. The gain-to-feed ratio increased linearly in d 7–14 (p = 0.06, tendency) and 14–21 postweaning (p<0.05). Fecal score linearly decreased in d 0–7, 7–14, and 14–21 (p<0.05) postweaning, resulting in a linear reduction in the overall period (p<0.05). Plasma malondialdehyde levels at d 28 postweaning showed a quadratic decrease with increasing MCFA levels with the lowest value at 0.8% (p<0.05). Plasma free fatty acid levels at d 14 postweaning linearly decreased with increasing MCFA levels (p<0.05). Fecal total short-chain fatty acid (SCFA) concentrations tended to be lower (p = 0.09, tendency) in the MCFA treatments (0.4%–1.6%) than the control treatment (0.0%). There were no differences in plasma superoxide dismutase activity and fecal ammonia concentrations among dietary treatments.

**Conclusion:**

Increasing MCFA supplementation levels up to 1.6% could improve growth rate, feed efficiency, and fecal consistency without affecting feed intake and have the potential to reduce oxidative stress and free fatty acid levels in weaning pigs, while fecal SCFA concentrations could be slightly reduced by MCFA supplementation.

## INTRODUCTION

Weaning causes severe stress to pigs, including inflammation, oxidative stress, and gut dysbiosis, leading to growth retardation and postweaning diarrhea. Feed consumption of newly weaned pigs is also limited in the first few days after weaning due to stress, leading to a negative energy balance and the mobilization of body lipid, which involves the catabolization of body fat [1]. Meeting the energy needs of weaning pigs is critical during the weaning transition period, and efficient energy sources are necessary to ensure a sufficient energy supply and improve their energy status after weaning.

In nursery diets, fat sources have been added to meet the energy requirements of weaning pigs. Although long-chain fatty acid (LCFA) sources such as soybean oil and beef tallow may have slightly higher energy content than medium-chain fatty acid (MCFA) sources like coconut oil, they play different physiological roles [2]. LCFAs are primarily used for energy storage whereas MCFAs, due to their shorter chain length (6–12 carbon atoms), are more readily utilized as an immediate and efficient energy source [3]. MCFAs do not require emulsification for digestion and absorption, are preferentially transported via the portal vein to the liver instead of the lymphatic system, and primarily undergo β-oxidation for energy production [3,4]. MCFAs are also involved in ketone body formation (*i.e*., acetoacetate, β-hydroxybutyrate), which serves as an instant energy substrate for the body [4]. These metabolic properties can make MCFAs particularly beneficial for young pigs with immature digestive systems, as they support efficient energy utilization, growth and development [3]. In addition to their role in energy metabolism, MCFAs have antibacterial and antiviral properties that can alleviate the negative impacts of disease infections in weaning pigs [5,6] and reduce the incidence of postweaning diarrhea under pathogen challenge [7,8]. Utilizing MCFAs in starter diets may improve pigs’ energy status and reduce postweaning health challenges in weaning pigs. In previous studies, Gebhardt et al [5] reported that MCFA supplementation of up to 1.5% enhanced the weaning pigs’ postweaning growth rate, feed intake, and feed efficiency. Thomas et al [9] also reported an enhanced growth rate and feed intake in nursery pigs with increasing MCFA supplementation levels of up to 2.0%. However, previous research with MCFA supplementation has predominantly focused on its effect on growth performance and fecal characteristics in weaned pigs [5,9,10], with lower supplementation levels (<0.5%) mainly investigated for their antibacterial and antiviral properties [7,8]. Limited information is available regarding its potential to improve energy status, antioxidant properties, fecal metabolites in weaning pigs. Additionally, their optimal supplementation levels during the weaning transition remain unclear. It is particularly important as weaning is a critical period characterized by stress, reduced feed intake leading to negative energy balance, increased oxidative stress and susceptibility to gut dysbiosis. Effective nutritional strategies, such as MCFA supplementation may help mitigate these challenges and support the health and performance of weaning during this transition.

Therefore, the objective of the current study was to demonstrate the effect of increasing MCFA supplementation levels up to 1.6% in the diets of newly weaned pigs on growth performance, fecal sore, antioxidant and energy status, and fecal metabolites.

## MATERIALS AND METHODS

### Animals, experimental design, and housing

A total of 100 newly weaned pigs (Camborough×PIC337 and [Camborough×Berkshire]×PIC337; initial body weight 6.68± 0.97 kg; weaned at 18.8±1.68 d of age) were allotted to 1 of 5 dietary treatments in 5 replicates with 4 pigs (2 barrows and 2 gilts) per pen based on body weight, breed, sex, and littermate in a randomized complete block design for a 28-d feeding trial. Treatments were based on increasing dietary MCFA supplementation levels from 0.0 (control), 0.4%, 0.8%, 1.2%, and 1.6%. A 50:50 blend (*w/w*) of caproic (C6:0) and caprylic acid (C8:0) in a free acid form (Excellentia International, Fairfield, NJ, USA) was used in the study. The 0.0% MCFA diet contained 2% of soybean oil, which was replaced with the MCFA blend (*w/w*) in the other MCFA-supplemented diets from 0.4% to 1.6%. As a result, the 1.6% MCFA treatment included 0.4% soybean oil to avoid potential negative impacts associated with the absence of LCFA sources in nursery diets such as deficiencies of essential fatty acids [11]. All pigs were housed in nursery pens (1.0 m×2.0 m) with woven-wire flooring and had free access to water and feed in an environmentally-controlled nursery facility. No creep feed was provided during the lactation period. The two diet phases included d 0–14 postweaning (Phase 1) and d 14–28 postweaning (Phase 2).

### Experimental diets

All pigs were fed corn-soybean meal-based mash diets formulated to meet or exceed nutrient requirement estimates of NRC [2] for pigs weighing 7–11 kg (Phase 1) and 11–25 kg (Phase 2; Table 1). A basal diet was first mixed without soybean oil to minimize variations in non-treatment diet components. This basal diet was then divided into five fractions. One fraction was mixed with 2% soybean oil without MCFA supplementation (control diet with 0.0% MCFA). For the remaining fractions, soybean oil was replaced by the assigned MCFA supplementation levels of 0.4%, 0.8%, 1.2%, and 1.6%, respectively, to create the MCFA-supplemented diets. Representative samples of mixed feed were collected at the feed mill and stored at −20°C until analyzed for chemical analysis.

### Data and sample collection

The pigs were individually weighed at the start of the trial, d 7, 14, 21, and 28 postweaning, and the pen-based feed disappearance was measured when the pigs were weighed to calculate average daily gain (ADG), average daily feed intake (ADFI), and gain-to-feed (G:F) ratio. The fecal score was recorded every day for the entire experimental period using a 4-scale fecal score system (1 = normal, 2 = soft, looser than normal feces, slight diarrhea, 3 = moderate diarrheic feces, and 4 = liquid, severe diarrhea) by observing individual pigs in each pen and assessing signs of stool consistency in the pen.

On d 14 and 28 postweaning, blood samples (10 mL) were collected from eight representative pigs (2 pigs per pen in the first 4 replicates; 1 barrow and 1 gilt per pen) selected based on average body weight in each pen. Samples were obtained via jugular venipuncture in disposable vacutainer tubes containing the anticoagulant K_3_ EDTA (Becton Dickinson, Franklin, NJ, USA). Plasma samples were obtained by centrifugation at 2,500×g for 30 min at 4°C and stored at −80°C until further analysis.

Fecal samples were collected from the same pigs used for blood collection by rectal palpation at d 14 and 28 postweaning, flash-frozen in liquid nitrogen, and stored at −80°C for fecal ammonia and short-chain fatty acid (SCFA) analysis.

### Chemical analysis

Feed samples were analyzed for dry matter (967.03) [12], crude protein (990.03) [12], ether extract (920.39) [12], neutral detergent fiber [13], acid detergent fiber [13], and chemical composition (Table 2).

Plasma samples were analyzed for antioxidant parameters, including superoxide dismutase (SOD; Cayman Chemical, Ann Arbor, MI, USA) activity and malondialdehyde (MDA; Cayman Chemical Company), and free fatty acids (Zen-Bio, Durham, NC, USA) levels using colorimetric kits and a spectrophotometer (Multiskan Skyhigh; Thermo Fisher Scientific, Waltham, MA, USA) following manufacturer’s instructions.

Fecal samples were analyzed for fecal SCFA concentrations by using the protocol described by Lourenco et al [14]. Briefly, fecal samples were solubilized in water at a 1:3 ratio (feces to water, *w/v*) and centrifuged at 10,000×g for 10 minutes. After centrifugation, 1 mL of the supernatant was transferred to a 2 mL tube, to which 0.2 mL of metaphosphoric acid (25% *w/v*) was added. The tube was then stored at −20°C overnight to freeze the sample. Following thawing, the samples were centrifuged again at 10,000×g for 10 minutes, and the resultant supernatant was transferred to polypropylene tubes. The supernatant was mixed with ethyl acetate in a 2:1 ratio (*v/v*), vortexed for 15 seconds, and then allowed to settle for 5 minutes. Subsequently, 0.5 mL of the top layer was transferred into screw-thread vials for the SCFA analysis. The SCFAs were quantified using a Shimadzu GC-2010 Plus gas chromatograph (Shimadzu, Kyoto, Japan) equipped with a flame ionization detector and a Zebron ZB-FFAP capillary column (30 m×0.32 mm×0.25 μm; Phenomenex, Torrance, CA, USA). The injection volume was 1.0 μL, and helium was used as carrier gas. The column temperature was initially set to 110°C and gradually increased to 200°C, with injector and detector temperatures set at 250°C and 350°C, respectively.

Fecal ammonia concentrations were measured using the phenol-hypochlorite colorimetric method described by Chaney and Marbach [15] with a GENESYS 30 spectrophotometer (Thermo Fisher Scientific). Briefly, fecal samples were solubilized in water at a 1:4 ratio (feces to water, *w/v*), then homogenized and centrifuged at 10,000×g for 10 minutes at 4°C. After centrifugation, 50 μL of the supernatant was mixed with 3 mL of phenol and 3 mL of hypochlorite reagents, followed by incubation at 39°C for 20 minutes to allow color development. After incubation, the absorbance was measured at 630 nm, and the ammonia concentration was determined using a standard calibration curve of ammonium chloride. The tubes containing the reaction mixture were incubated at 39°C for 20 minutes to allow the color development. After incubation, the absorbance was measured at 630 nm, and the ammonia concentration was determined based on a standard calibration curve of ammonium chloride.

### Statistical analysis

All data obtained in the current study were analyzed following a randomized complete block design using the PROC MIXED procedure of SAS (ver. 9.4; SAS Institute, Cary, NC, USA). A pen was used as an experimental unit to analyze growth performance data. An individual pig was used as an experimental unit for blood and fecal analyses. The models included the treatment as a fixed effect and the replicate as a random effect for growth performance and the replicate within pen and pen as random effects for blood and fecal parameters. The least-square means were separated using the PDIFF option in SAS. Orthogonal polynomial contrast analysis was performed to evaluate the linear and quadratic effects of increasing MCFA supplementation levels. A single degree of freedom contrast was performed to make the comparison between control treatment vs. combined MCFA treatments (MCFA supplementation levels of 0.4%, 0.8%, 1.2%, and 1.6%). Statistical differences were established at p≤0.05, and tendencies were established at p≤0.10.

## RESULTS

### Diet analysis

Although there were some variations in proximate analysis, the chemical composition was similar across dietary treatments in each phase, as the basal diet was used for all treatment diets (Table 2). However, the ether extract content decreased with increasing levels of MCFA supplementation, showing 1.49% and 1.29% differences between 0.0% and 1.6% MCFA diets in Phase 1 and 2, respectively, which were consistent with the levels of MCFA supplementation.

### Growth performance and fecal score

There was no difference in body weight until d 14 postweaning (p>0.10; Table 3). However, increasing MCFA supplementation levels tended to increase body weight at d 21 and 28 postweaning (p<0.07). The ADG tended to increase linearly in d 7-14 postweaning (p = 0.10) and overall period (p = 0.06) with significant increases observed in d 14–21 and 14–28 postweaning (p<0.05). The ADFI decreased quadratically with increasing MCFA supplementation levels in d 0–7 postweaning (p<0.05) with the lowest value at the 1.2% MCFA level, while it tended to increase quadratically in d 14–21 postweaning (p = 0.10) with the greatest value at the 0.4% MCFA level. The G:F ratio tended to increase linearly with increasing MCFA supplementation levels in d 7–14 postweaning (p = 0.06) and increased significantly in d 14–21 postweaning (p<0.05).

Compared with the control group, the MCFA treatments (0.4% to 1.6% MCFA treatments) tended to have greater body weight at d 21 postweaning (p = 0.06), ADG in d 7–14 postweaning (p = 0.10) and G:F ratio in d 7–14 postweaning (p = 0.07), significantly increased ADG and ADFI in d 14–21 postweaning (p<0.05) and tended to reduce ADFI in d 0–7 postweaning (p = 0.08).

Fecal score linearly decreased with increasing MCFA supplementation levels until d 21 postweaning (p<0.05; Table 4), resulting in linear decreases in fecal score in all phases and the overall period (p<0.05). The MCFA treatments consistently had lower fecal scores than the control treatment in Phase 1, Phase 2, and overall periods (p<0.05) except for d 21–28 postweaning.

### Plasma antioxidant status and free fatty acid levels

There was no significant difference in plasma SOD activity among dietary treatments (Table 5), while plasma MDA levels showed a quadratic decrease with increasing MCFA supplementation levels (p<0.05), with the lowest value at the 0.8% MCFA level.

Plasma-free fatty acid levels at d 14 postweaning linearly decreased with increasing MCFA supplementation levels (p<0.05), and the control treatment tended to have greater plasma-free fatty acid levels than the MCFA treatments (p = 0.06), while there was no significant difference at d 28 postweaning (Table 6).

### Fecal short-chain fatty acid and ammonia concentrations

There were no significant differences in fecal SCFA concentrations at d 14 and 28 postweaning with increasing MCFA supplementation levels (p>0.10; Table 7). At d 28 postweaning, the MCFA treatments had significantly lower valerate (p< 0.05) concentration and tended to have lower propionate (p = 0.08) and total SCFA (p = 0.09) concentrations compared with the control treatment. In the result of fecal SCFA composition as a percentage of total SCFA (Table 8), there were no significant differences at d 28 postweaning, while caproate composition tended to show a quadratic response (p = 0.08), with the lowest value at 1.2% MCFA levels. There was no significant difference in fecal ammonia concentrations at d 14 and 28 postweaning among dietary treatments (p>0.10; Table 9).

## DISCUSSION

MCFAs, which are saturated fatty acids with carbon chain lengths ranging from 6 to 12, are known for their antibacterial and antiviral properties. As a result, MCFAs are often added to nursery diets to help reduce postweaning diarrhea and enhance growth performance in pigs [6]. Additionally, MCFAs provide a rapid source of energy for pigs experiencing a negative energy balance. While LCFAs, such as vegetable oils and animal fats, are typically used in nursery diets [3,4], MCFAs are absorbed and utilized more efficiently for energy due to their shorter carbon chains. Although previous studies have shown that MCFA supplementation can reduce postweaning diarrhea by reducing pathogenic bacteria in the gut [7,8], and enhance growth performance [16,17], there is limited information on how higher MCFA supplementation levels affect not only growth but also antioxidant status, energy balance, and fecal metabolites. Moreover, the optimal MCFA supplementation levels in nursery diets has yet to be established. Therefore, this study evaluated the effect of increasing MCFA supplementation levels up to 1.6% on growth performance, fecal score, antioxidant and energy status, and fecal metabolites in nursery pigs.

In the diet analysis, ether extract content decreased with increasing MCFA supplementation levels in each phase, which agreed with Thomas et al [9]. The difference in ether extract content across treatments aligned with the differences in dietary MCFA supplementation levels. In Phase 1 and 2, ether extract content for the 0.0% MCFA supplementation was 4.50% and 4.18%, respectively. In contrast, at the 1.6% MCFA supplementation level, ether extract content was 3.01% and 2.89%, showing a difference of 1.49% and 1.29%, respectively. In the current study, a single basal diet was used to prepare all treatment diets in each phase, with soybean oil only being replaced by MCFA on a weight-to-weight basis. As a result, the total fatty acid content should remain consistent across dietary treatments. However, the result of the ether extract analysis showed a decrease in its levels with increasing MCFA supplementation levels, suggesting that ether extraction may not have fully detected the MCFAs in the diets. MCFAs are shorter-chain fatty acids than those found in vegetable oils and animal fats, making them more polar and giving them a greater affinity for water molecules than longer-chain fatty acids [3,4]. Due to their relatively greater polarity, MCFAs may not be as effectively extracted by the ether extraction process, especially when compared to non-polar lipids like triglycerides, which are more readily dissolved in organic solvents [9]. Consequently, measurements that rely on ether extract content in diets (e.g., digestibility or energy calculation using ether extract in equations) may be inaccurate when testing the effect of dietary MCFA supplementation in animals due to incomplete extraction during analysis.

In the current study, increasing MCFA supplementation levels up to 1.6% by replacing soybean oil improved postweaning growth performance. Growth rate linearly increased in d 7–14 and d 14–21 postweaning, leading to linear increases in growth rate in d 14–28 postweaning and the overall period and in body weight at d 21 and 28 postweaning. Feed efficiency also linearly increased significantly in d 14–21 postweaning and d 7–14 postweaning. This result agrees with previous studies reporting that dietary MCFA supplementation up to 1.5%–2.0% increased postweaning growth rate, feed intake, and feed efficiency of weaning pigs [5,9]. Newly weaned pigs had a negative energy balance due to weaning stress and downregulated fatty acid transporters and chylomicron synthesis [1,18], which is critical for lipid absorption and utilization. However, MCFAs are known to be absorbed from the intestine faster than LCFAs and transported via the portal vein instead of the lymphatic vessels through which LCFAs are transported [4]. The shorter carbon chain lengths of caproic (C6:0) and caprylic (C8:0) acids, along with a lower melting point and higher solubility compared with LCFAs, result in less dependence on bile during digestion and absorption processes [3]. The MCFAs also pass through the mitochondrial membrane in hepatic cells to be used for energy production [4], which is a quicker and more efficient process compared with LCFAs, which require carnitine acyltransferase to pass through the mitochondrial membrane [19]. In addition, after β-oxidation, the MCFAs can increase the level of energy substrates in the form of ketone bodies that can be used for rapid energy production [3]. The results of the current study indicate that MCFA supplementation could improve postweaning growth rate and have the potential to improve feed efficiency in pigs up to a 1.6% supplementation level.

In the current study, a free acid form of MCFA did not reduce feed intake of pigs, while Cera et al [20] showed that a free form of MCFA reduced feed intake. Additionally, feed intake in d 14–21 postweaning tended to increase quadratically with increasing MCFA supplementation levels, resulting in greater feed intake in the MCFA treatments compared to the control treatment. This suggests that supplementing up to 1.6% MCFA in a free acid form did not negatively impact feed consumption in weaning pigs, even though the free acid forms of MCFA have a pungent odor, which could potentially reduce the feed preference of pigs [3]. To mitigate the potential negative impact in feed consumption, commercially available MCFA products are typically in the form of mono-, di-, or triglycerides or are encapsulated with lipids and carbohydrates, which help reduce their smell and slow their absorption in the stomach, allowing the MCFAs to be gradually released in the small intestine for their antibactieral effects in the intestine [6]. However, the free fatty acid forms of MCFAs have advantages over their corresponding acylglycerol forms because acylglycerols require digestion by lipase in the small intestine to be activated for their antibacterial properties. In contrast, the free acid form can be instantly utilized in the small intestine [6].

The fecal score decreased linearly with increasing MCFA supplementation levels in the first 3 weeks of the nursery period, resulting in a linear decrease in the overall period, although there was no difference in d 21–28 postweaning. The antibacterial and antiviral effects of MCFA can alter the gut environment by destabilizing the bacterial cell membrane, inhibiting bacterial lipases, activating bacterial autolytic enzymes, and lowering the pH, resulting in the inactivation of cytoplasmic enzymes in bacteria cells [3,5,21]. Previous studies reported that MCFA supplementation as a blend of organic acids could reduce the incidence of diarrhea when challenged with pathogenic bacteria [7,22] and reduce the colonization of pathogenic bacteria in the intestine of weaning pigs [8,21]. Matsui et al [21] reported that MCFA supplementation reduced the count of *Escherichia coli* and *Campylobacter jejuni* in feces of pigs compared to the control diet. López-Colom et al [8] also reported that MCFA supplementation reduced the pathogenic bacteria count in the digesta of pigs challenged with *Salmonella* or ETEC F4. In addition, it has been reported that MCFA supplementation could modify intestinal microbiota [17,20]. This result suggests that the MCFA supplementation could improve fecal consistency, suggesting that pigs may have a more desirable gut environment, resulting in improved growth performance.

Plasma SOD activity was not different among dietary treatments, while plasma MDA levels decreased with increasing MCFA supplementation levels up to 0.8% at d 28 postweaning, indicating a potential antioxndant response. This is contrast with previous studies reporting more pronounced effects of MCFA supplementation on antioxidant status in pigs. Lee and Kang [23] reported that the pigs fed diets containing 0.5% capric acid (C10:0) reduced serum TNF-α, IL-6, and MDA levels and increased both SOD and glutathione peroxidase activities [23]. Similarly, Jiao et al [17] reported that 0.6% MCFA (31% caprylic acid [C8:0], 22% capric acid [C10:0], and 6.8% lauric acid [C12:0]) supplementation reduced MDA levels and enhanced antioxidant enzyme activities in weaning pigs. Long et al [16] reported that the 0.2% MCFA-organic acid blends decreased serum hydroxyl radicals. This difference in antioxidant responses between the current and previous studies may be attributed to the differences in MCFA composition, dosage, feeding duratioin, or the physiological state of the pigs. Unlike previous studies using relatively low levels of MCFA, the current study examined higher supplementation levels up to 1.6%, that has not been extensively evaluated for antioxidant effects. These findings suggest that while MCFAs may help mitigate oxidative stress in weaning pigs, their effectiveness likely depends on the dosage, MCFA profile, and the health or stress status of pigs.

However, higher levels of MCFA supplementation may not provide additional benefits in reducing oxidative stress in pigs, as plasma MDA levels increased at 1.2% and 1.6% MCFA supplementation levels compared to 0.4% and 0.8% levels, although plasma MDA levels remained numerically lower than those in the 0.0% MCFA supplementation group. Miyagawa et al [24] reported that increased accumulation of lipoperoxidation markers was observed in cardiomyocytes when excessive amounts of lauric acid (C12:0; 5% and 10%) were supplemented in the diets of mice, compared to the 2% supplementation level, suggesting that high concentrations of MCFAs might increase oxidative stress. This may be because MCFAs can be directly passed into mitochondria, where they undergo β-oxidation, leading to excessive oxidative phosphorylation, resulting in oxidative stress. It should be noted that although the growth rate increased linearly with increasing MCFA supplementation levels up to 1.6%, the potential impact on health should be considered when the optimum supplementation levels of MCFAs are determined.

Plasma-free fatty acid levels at d 14 postweaning decreased linearly with increasing MCFA supplementation levels, and the MCFA treatments tended to have lower plasma-free fatty acid levels than the control treatments. This result agreed with Świątkiewicz et al [25], reporting that 0.3% of MCT oil mainly containing caprylic (C8:0; 61%) and capric (C10:0; 39%) acids or 0.3% of caprylic acid (C8:0) supplementation could slightly reduce serum free fatty acid levels of pigs at d 60 of age. At weaning, pigs may experience a negative energy balance due to the transition to a solid diet, limited feed intake, and reduced lipid absorption, as gene expression related to fatty acid transport and chylomicron synthesis decreases after weaning, leading to increased body fat catabolism and resultant high free fatty acid levels to supply energy [18,26]. This result suggests that MCFA supplementation could reduce body fat mobilization in pigs, as MCFAs serve as an efficient energy source for weaning pigs, which may explain the improved growth rate and feed efficiency in the current study.

The MCFA supplementation did not affect fecal ammonia content at d 14 and 28 postweaning. This result agreed with López-Colom et al [8], reporting that MCFA supplementation did not affect ileal and colonic ammonia concentrations in weaning pigs challenged with *Salmonella* and ETEC F4 although it reduced the count of pathogenic bacteria in the digesta. Although increasing MCFA supplementation levels had no significant effect on fecal SCFA concentrations and composition, the MCFA treatments (0.4% to 1.6% supplementation levels) showed slightly lower propionate and valerate concentrations, as well as numerically lower concentrations of the other SCFA, resulting in a slight reduction in total SCFA concentrations in feces compared to the control treatment at d 28 postweaning. This result agreed with Świątkiewicz et al [25], reporting that MCT oil supplementation to piglets reduced propionate, butyrate, valerate, and total SCFA concentrations in the cecum, although caprylic acid (C8:0) supplementation had no effect. Although there is no clear explanation, this result suggests that MCFA supplementation may influence the gut environment and microflora, thereby affecting fecal consistency and SCFA production. Jiao et al [17] reported that the MCFA supplementation at 0.06% for weaning pigs decreased colonic isobutyrate and isovalerate concentrations, which could improve intestinal barrier functions, although there was no significant difference observed in the other SCFA concentrations. In contrast, Long et al [16] reported that supplementation of organic acid-containing MCFA in nursery pig diets increased the concentrations of acetate, propionate, butyrate, and isobutyrate in feces, resulting in increased total SCFA concentrations. In addition, it has been reported that MCFA supplementation had no significant effect on fecal SCFA concentrations in pigs, although it could reduce pathogenic bacteria [21]. Given the inconsistent effects of MCFA supplementation in SCFA production in the gut, despite its antibacterial activity that can alter gut microbiota [16,17], further studies are needed to clearly demonstrate how the fecal SCFA concentrations are correlated with changes in gut microbiota when MCFAs are supplemented at higher levels.

## CONCLUSION

Increasing MCFA supplementation levels up to 1.6% could improve growth rate, feed efficiency, and fecal consistency without affecting feed intake. The MCFA supplementation also showed the potential to reduce oxidative stress when supplemented up to 0.8% in diets and decreased blood free fatty acid levels in weaning pigs. Although there was no virtual effect, the fecal SCFA concentrations could be slightly reduced by the MCFA supplementation.

## Figures and Tables

**Table 1:** Diet formulation and calculated chemical composition

Ingredients (%)	Phase 1 d 0–14 postweaning	Phase 2 d 14–28 postweaning
Corn	40.89	50.40
Soybean meal (48% crude protein)	26.50	31.50
Whey, dried	10.00	7.50
Oats	2.50	2.50
HP300^1)^	5.00	2.00
Lactose	5.00	0.00
Fish meal	2.50	1.50
Animal plasma	3.00	0.00
Soybean oil	2.00	2.00
L-Lysine·HCl	0.12	0.22
DL-Methionine	0.16	0.15
L-Threonine	0.09	0.13
Dicalcium phosphate	0.64	0.55
Limestone	1.00	0.95
Salt	0.25	0.25
Vitamin mix^2)^	0.20	0.20
Trace mineral mix^3)^	0.15	0.15
Calculated chemical composition		
Metabolizable energy (kcal/kg)	3,415	3,390
Crude protein (%)	24.53	22.85
SID lysine (%)	1.40	1.28
SID methionine+cysteine (%)	0.85	0.77
Total Ca (%)	0.80	0.70
STTD P (%)	0.40	0.33

1) Hamlet Protein, Findlay, OH, USA.

2) The vitamin premix supplied the following per kilogram of diet: 11,000 IU of vitamin A, 1,600 IU of vitamin D_3_, 99 IU of vitamin E, 4.4 mg of vitamin K, 55 μg of vitamin B_12_, 9.9 mg of riboflavin, 31.9 mg of pantothenic acid, 55 mg of niacin, 0.9 mg of folic acid, 3.9 mg of vitamin B_6_, 3.1 mg of thiamin, and 0.3 mg of biotin, 600 mg of choline chloride.

3) The trace mineral premix supplied the following per kilogram of diet: 33 mg of Mn as manganous oxide, 110 mg of Fe as ferrous sulfate, 110 mg of Zn as zinc sulfate, 16.5 mg of Cu as copper sulfate, 0.3 mg of I as Ca iodate, 0.3 mg of Se as sodium selenite.

SID, standardized ileal digestible; STTD, standardized total tract digestible.

**Table 2:** Chemical composition of experimental diets (%) containing increasing levels of medium-chain fatty acids (MCFA) in each phase

Item	Treatment^1)^				

	0 (control)	0.4	0.8	1.2	1.6
Phase 1 (d 0–14 postweaning)					
Dry matter	90.88	89.95	89.70	89.48	89.11
Crude protein	25.44	26.66	26.61	26.66	26.21
Ether extract	4.50	3.79	3.68	3.31	3.01
Neutral detergent fiber	7.27	7.16	7.07	6.21	6.60
Acid detergent fiber	2.73	2.71	2.64	2.60	2.45
Phase 2 (d 14–28 postweaning)					
Dry matter	89.70	89.35	88.72	88.41	88.37
Crude protein	22.78	24.11	24.82	22.85	23.60
Ether extract	4.18	3.87	3.69	3.36	2.89
Neutral detergent fiber	8.63	8.26	8.76	8.25	8.84
Acid detergent fiber	3.18	2.81	2.91	2.96	3.03

1) Treatments: 0.0%, 0.4%, 0.8%, 1.2%, and 1.6% of MCFA supplementation level. The MCFA was a 50:50 blend of caproic (C6:0) and caprylic acid (C8:0) in a free acid form. The 0.0% MCFA diet contained 2% soybean oil. It was replaced with MCFA at the assigned levels on a weight-to-weight basis.

**Table 3:** Postweaning growth performance of pigs fed diets supplemented with increasing levels of medium-chain fatty acids (MCFA) in postweaning period

Item	Treatment^1)^					SEM	p-values		
	
	0 (control)	0.4	0.8	1.2	1.6		Linear	Quadratic	Control vs. MCFA^2)^
Body weight (kg)									
d 0	6.70	6.74	6.65	6.71	6.66	0.45	0.55	0.80	0.95
d 7	7.13	7.24	7.02	6.89	7.13	0.47	0.21	0.29	0.53
d 14	8.63	8.97	8.67	8.70	8.96	0.55	0.45	0.75	0.29
d 21	11.58	12.28	12.02	12.34	12.52	0.76	0.07	0.70	0.06
d 28	15.88	16.38	16.43	16.53	17.18	1.05	0.06	0.83	0.13
ADG (kg/d)									
d 0–7	0.063	0.072	0.053	0.024	0.067	0.01	0.36	0.25	0.56
d 7–14	0.214	0.248	0.235	0.259	0.262	0.02	0.10	0.74	0.10
d 14–21	0.421	0.472	0.480	0.520	0.508	0.04	0.04	0.45	0.05
d 21–28	0.614	0.586	0.630	0.599	0.665	0.05	0.28	0.36	0.87
d 0–14 (Phase I)	0.139	0.160	0.144	0.142	0.164	0.01	0.33	0.68	0.26
d 14–28 (Phase II)	0.518	0.529	0.555	0.559	0.587	0.04	0.05	0.90	0.17
d 0–28 (overall)	0.328	0.344	0.349	0.351	0.376	0.02	0.06	0.81	0.14
ADFI (kg/d)									
d 0–7	0.164	0.156	0.145	0.139	0.160	0.01	0.24	0.02	0.08
d 7–14	0.344	0.366	0.346	0.380	0.364	0.02	0.32	0.74	0.30
d 14–21	0.609	0.706	0.676	0.678	0.662	0.04	0.42	0.10	0.05
d 21–28	1.139	1.210	1.120	1.216	1.160	0.07	0.83	0.80	0.65
d 0–14 (Phase I)	0.254	0.261	0.245	0.260	0.262	0.01	0.65	0.54	0.80
d 14–28 (Phase II)	0.874	0.958	0.898	0.947	0.911	0.04	0.63	0.40	0.25
d 0–28 (overall)	0.564	0.610	0.571	0.603	0.586	0.02	0.61	0.54	0.29
G:F									
d 0–7	0.343	0.455	0.356	0.166	0.408	0.08	0.50	0.55	0.97
d 7–14	0.618	0.674	0.672	0.678	0.717	0.03	0.06	0.83	0.07
d 14–21	0.698	0.665	0.707	0.762	0.768	0.04	0.04	0.48	0.48
d 21–28	0.539	0.511	0.569	0.503	0.585	0.06	0.54	0.55	0.95
d 0–14 (Phase I)	0.536	0.609	0.580	0.542	0.628	0.03	0.24	0.88	0.13
d 14–28 (Phase II)	0.591	0.565	0.619	0.596	0.649	0.05	0.20	0.54	0.68
d 0–28 (overall)	0.579	0.572	0.610	0.584	0.644	0.04	0.17	0.56	0.51

n = 5 replicate pens per treatment.

1) Treatments: 0.0%, 0.4%, 0.8%, 1.2%, and 1.6% of MCFA supplementation level. The MCFA was a 50:50 blend of caproic (C6:0) and caprylic acid (C8:0) in a free acid form. The 0.0% MCFA diet contained 2% soybean oil. It was replaced with MCFA at the assigned levels on a weight-to-weight basis.

2) Single degree of freedom contrast (Control [0.0% MCFA] vs. combined MCFA treatments from 0.4% to 1.6% supplementation levels).

SEM, standard error of the means; ADG, average daily gain; ADFI, average daily feed intake; G:F, gain-to-feed.

**Table 4:** Fecal score of pigs fed diets supplemented with increasing levels of medium-chain fatty acids (MCFA) in postweaning period

Item	Treatment^1)^					SEM	p-values		
	
	0 (control)	0.4	0.8	1.2	1.6		Linear	Quadratic	Control vs. MCFA^2)^
d 0–7	1.59	1.48	1.17	1.30	1.14	0.09	0.01	0.28	0.01
d 7–14	1.91	1.51	1.20	1.52	1.11	0.15	0.01	0.29	0.01
d 14–21	1.22	1.11	1.00	1.14	1.00	0.06	0.04	0.40	0.03
d 21–28	1.07	1.10	1.00	1.00	1.00	0.05	0.18	0.84	0.46
d 0–14 (Phase I)	1.75	1.50	1.18	1.41	1.13	0.11	0.01	0.25	0.01
d 14–28 (Phase II)	1.15	1.11	1.00	1.07	1.00	0.04	0.02	0.44	0.03
d 0–28 (overall)	1.45	1.30	1.09	1.24	1.06	0.07	0.01	0.25	0.01

n = 5 replicate pens per treatment.

Fecal score was measured using a 4-scale fecal score system (1 = normal, 2 = soft, looser than normal feces, slight diarrhea, 3 = moderate diarrheic feces, and 4 = liquid, severe diarrhea) by observing individual pigs in each pen and assessing signs of stool consistency in the pen.

1) Treatments: 0.0%, 0.4%, 0.8%, 1.2%, and 1.6% of MCFA supplementation level. The MCFA was a 50:50 blend of caproic (C6:0) and caprylic acid (C8:0) in a free acid form. The 0.0% MCFA diet contained 2% soybean oil. It was replaced with MCFA at the assigned levels on a weight-to-weight basis.

2) Single degree of freedom contrast (Control [0.0% MCFA] vs. combined MCFA treatments from 0.4% to 1.6% supplementation levels).

SEM, standard error of the means.

**Table 5:** Plasma superoxide dismutase activity and malondialdehyde level in pigs fed diets supplemented with increasing levels of medium-chain fatty acids (MCFA) in postweaning period

Item	Treatment^1)^					SEM	p-values		
	
	0 (control)	0.4	0.8	1.2	1.6		Linear	Quadratic	Control vs. MCFA^2)^
Superoxide dismutase (U/mL)									
d 14	6.06	5.99	5.93	5.32	5.59	0.48	0.29	0.94	0.51
d 28	5.30	5.73	4.99	4.69	5.95	0.51	0.86	0.23	0.94
Malondialdehyde (μM)									
d 14	13.02	14.21	13.99	14.71	13.86	1.50	0.63	0.56	0.46
d 28	14.13	10.67	9.32	13.02	13.26	1.63	0.90	0.04	0.15

n = 8 per treatment.

1) Treatments: 0.0%, 0.4%, 0.8%, 1.2%, and 1.6% of MCFA supplementation level. The MCFA was a 50:50 blend of caproic (C6:0) and caprylic acid (C8:0) in a free acid form. The 0.0% MCFA diet contained 2% soybean oil. It was replaced with MCFA at the assigned levels on a weight-to-weight basis.

2) Single degree of freedom contrast (Control [0.0% MCFA] vs. combined MCFA treatments from 0.4 to 1.6% supplementation levels).

SEM, standard error of the means.

**Table 6:** Plasma free fatty acid levels in pigs fed diets supplemented with increasing levels of medium-chain fatty acids (MCFA) in postweaning period

Item	Treatment^1)^					SEM	p-values		
	
	0 (control)	0.4	0.8	1.2	1.6		Linear	Quadratic	Control vs. MCFA^2)^
Free fatty acid (μM)									
d 14	146.65	140.48	128.57	98.35	114.01	12.85	0.01	0.58	0.06
d 28	85.55	78.60	84.89	82.94	86.73	7.87	0.74	0.59	0.75

n = 8 per treatment.

1) Treatments: 0.0%, 0.4%, 0.8%, 1.2%, and 1.6% of MCFA supplementation level. The MCFA was a 50:50 blend of caproic (C6:0) and caprylic acid (C8:0) in a free acid form. The 0.0% MCFA diet contained 2% soybean oil. It was replaced with MCFA at the assigned levels on a weight-to-weight basis.

2) Single degree of freedom contrast (Control [0.0% MCFA] vs. combined MCFA treatments from 0.4% to 1.6% supplementation levels).

SEM, standard error of the means.

**Table 7:** Fecal short-chain fatty acid (SCFA) concentrations (mM) in pigs fed diets supplemented with increasing levels of medium-chain fatty acids (MCFA) in postweaning period

Item	Treatment^1)^					SEM	p-values		
	
	0 (control)	0.4	0.8	1.2	1.6		Linear	Quadratic	Control vs. MCFA^2)^
d 14									
Acetate	35.54	37.82	34.23	36.12	35.50	2.93	0.80	0.97	0.87
Propionate	15.35	16.15	14.46	13.24	16.28	1.99	0.84	0.43	0.86
Isobutyrate	0.64	0.62	0.65	0.62	0.77	0.12	0.52	0.56	0.84
Butyrate	6.68	7.15	5.47	5.72	6.63	1.03	0.61	0.42	0.66
Isovalerate	0.25	0.24	0.25	0.25	0.30	0.06	0.55	0.51	0.93
Valerate	2.70	3.21	2.62	2.46	3.31	0.55	0.80	0.59	0.74
Caproate	1.11	1.20	0.86	0.60	1.59	0.34	0.74	0.14	0.89
Total SCFA	62.27	66.39	58.55	58.95	64.38	6.08	0.84	0.55	0.97
d 28									
Acetate	24.17	19.98	23.29	21.57	21.88	2.14	0.55	0.51	0.17
Propionate	15.44	10.97	13.82	11.60	12.88	1.56	0.37	0.28	0.08
Isobutyrate	0.43	0.40	0.34	0.38	0.36	0.08	0.50	0.71	0.49
Butyrate	5.86	4.55	4.49	5.01	4.35	0.74	0.29	0.50	0.14
Isovalerate	0.17	0.15	0.14	0.15	0.14	0.03	0.50	0.75	0.50
Valerate	2.94	1.92	2.37	1.70	1.95	0.43	0.11	0.38	0.05
Caproate	1.36	1.19	1.19	0.92	1.12	0.31	0.45	0.69	0.47
Total SCFA	50.37	39.16	45.64	41.34	42.68	4.15	0.32	0.36	0.09

n = 8 per treatment.

1) Treatments: 0.0%, 0.4%, 0.8%, 1.2%, and 1.6% of MCFA supplementation level. The MCFA was a 50:50 blend of caproic (C6:0) and caprylic acid (C8:0) in a free acid form. The 0.0% MCFA diet contained 2% soybean oil. It was replaced with MCFA at the assigned levels on a weight-to-weight basis.

2) Single degree of freedom contrast (Control [0.0% MCFA] vs. combined MCFA treatments from 0.4% to 1.6% supplementation levels).

SEM, standard error of the means.

**Table 8:** Fecal shorth-chain fatty acid (SCFA) composition (% of total SCFA) in pigs fed diets supplemented with increasing levels of medium-chain fatty acids (MCFA) in postweaning period

Item	Treatment^1)^					SEM	p-values		
	
	0 (control)	0.4	0.8	1.2	1.6		Linear	Quadratic	Control vs. MCFA^2)^
d 14									
Acetate	57.18	58.35	58.76	62.99	55.99	2.37	0.77	0.17	0.48
Propionate	24.32	23.83	24.50	21.19	24.98	1.37	0.76	0.37	0.63
Isobutyrate	1.08	0.97	1.14	1.03	1.11	0.19	0.84	0.88	0.91
Butyrate	10.62	10.32	9.23	9.27	10.14	0.83	0.46	0.28	0.34
Isovalerate	0.44	0.36	0.44	0.42	0.43	0.09	0.88	0.74	0.73
Valerate	4.46	4.60	4.47	3.90	4.88	0.73	0.95	0.62	1.00
Caproate	1.88	1.56	1.47	1.09	2.36	0.45	0.74	0.08	0.57
d 28									
Acetate	49.39	52.24	51.84	51.76	52.41	2.93	0.48	0.66	0.34
Propionate	30.09	28.12	29.71	28.82	30.00	2.01	0.91	0.48	0.57
Isobutyrate	0.85	0.99	0.78	0.92	0.80	0.14	0.70	0.74	0.87
Butyrate	11.27	10.99	9.69	11.78	9.96	0.83	0.48	0.92	0.47
Isovalerate	0.33	0.38	0.31	0.35	0.31	0.06	0.71	0.74	0.93
Valerate	5.52	4.64	5.05	4.19	4.18	0.63	0.12	0.84	0.16
Caproate	2.56	2.65	2.62	2.18	2.35	0.57	0.63	0.90	0.87

n = 8 per treatment.

1) Treatments: 0.0%, 0.4%, 0.8%, 1.2%, and 1.6% of MCFA supplementation level. The MCFA was a 50:50 blend of caproic (C6:0) and caprylic acid (C8:0) in a free acid form. The 0.0% MCFA diet contained 2% soybean oil. It was replaced with MCFA at the assigned levels on a weight-to-weight basis.

2) Single degree of freedom contrast (Control [0.0% MCFA] vs. combined MCFA treatments from 0.4% to 1.6% supplementation levels).

SEM, standard error of the means.

**Table 9:** Fecal ammonia concentrations (mM) in pigs fed diets supplemented with increasing levels of medium-chain fatty acids (MCFA) in postweaning period

Item	Treatment^1)^					SEM	p-values		
	
	0 (control)	0.4	0.8	1.2	1.6		Linear	Quadratic	Control vs. MCFA^2)^
Ammonia (mM)									
d 14	6.74	4.87	5.31	4.55	5.84	1.23	0.56	0.23	0.19
d 28	4.73	3.84	3.71	4.85	3.57	0.99	0.64	0.88	0.46

n = 8 per treatment.

1) Treatments: 0.0%, 0.4%, 0.8%, 1.2%, and 1.6% of MCFA supplementation level. The MCFA was a 50:50 blend of caproic (C6:0) and caprylic acid (C8:0) in a free acid form. The 0.0% MCFA diet contained 2% soybean oil. It was replaced with MCFA at the assigned levels on a weight-to-weight basis.

2) Single degree of freedom contrast (Control [0.0% MCFA] vs. combined MCFA treatments from 0.4% to 1.6% supplementation levels).

SEM, standard error of the means.

## References

[1] van Kempen TATG, Hulshof TG, Gerrits WJJ, Zijlstra RT (2023). Review: the amazing gain-to-feed ratio of newly weaned piglets: sign of efficiency or deficiency?. animal.

[2] National Research Council (2012). Nutrient requirements of swine.

[3] Zentek J, Buchheit-Renko S, Ferrara F, Vahjen W, Van Kessel AG, Pieper R (2011). Nutritional and physiological role of medium-chain triglycerides and medium-chain fatty acids in piglets. Anim Health Res Rev.

[4] Odle J (1997). New insights into the utilization of medium-chain triglycerides by the neonate: observations from a piglet model. J Nutr.

[5] Gebhardt JT, Thomson KA, Woodworth JC (2019). Effect of dietary medium-chain fatty acids on nursery pig growth performance, fecal microbial composition, and mitigation properties against porcine epidemic diarrhea virus following storage. J Anim Sci.

[6] Jackman JA, Boyd RD, Elrod CC (2020). Medium-chain fatty acids and monoglycerides as feed additives for pig production: towards gut health improvement and feed pathogen mitigation. J Anim Sci Biotechnol.

[7] He Y, Jinno C, Li C (2022). Effects of a blend of essential oils, medium-chain fatty acids, and a toxin-adsorbing mineral on diarrhea and gut microbiome of weanling pigs experimentally infected with a pathogenic Escherichia coli. J Anim Sci.

[8] López-Colom P, Castillejos L, Rodríguez-Sorrento A, Puyalto M, Mallo JJ, Martín-Orúe SM (2019). Efficacy of medium-chain fatty acid salts distilled from coconut oil against two enteric pathogen challenges in weanling piglets. J Anim Sci Biotechnol.

[9] Thomas LL, Woodworth JC, Tokach MD (2020). Evaluation of different blends of medium-chain fatty acids, lactic acid, and monolaurin on nursery pig growth performance. Transl Anim Sci.

[10] Dahmer PL, Leubcke GE, Lerner AB, Jones CK (2020). Effects of medium-chain fatty acids as alternatives to ZnO or antibiotics in nursery pig diets. Transl Anim Sci.

[11] Jadhav HB, Annapure US (2023). Triglycerides of medium-chain fatty acids: a concise review. J Food Sci Technol.

[12] Association of Official Analytical Chemists (AOAC) (2006). International Official methods of analysis.

[13] Van Soest PJ, Robertson JB, Lewis BA (1991). Methods for dietary fiber, neutral detergent fiber, and nonstarch polysaccharides in relation to animal nutrition. J Dairy Sci.

[14] Lourenco JM, Kieran TJ, Seidel DS (2020). Comparison of the ruminal and fecal microbiotas in beef calves supplemented or not with concentrate. PLOS ONE.

[15] Chaney AL, Marbach EP (1962). Modified reagents for determination of urea and ammonia. Clin Chem.

[16] Long SF, Xu YT, Pan L (2018). Mixed organic acids as antibiotic substitutes improve performance, serum immunity, intestinal morphology and microbiota for weaned piglets. Anim Feed Sci Technol.

[17] Jiao S, Zheng Z, Zhuang Y, Tang C, Zhang N (2023). Dietary medium-chain fatty acid and Bacillus in combination alleviate weaning stress of piglets by regulating intestinal microbiota and barrier function. J Anim Sci.

[18] He Y, Liu N, Ji Y, Tso P, Wu Z (2022). Weaning stress in piglets alters the expression of intestinal proteins involved in fat absorption. J Nutr.

[19] Yen HC, Lai WK, Lin CS, Chiang SH (2015). Medium-chain triglyceride as an alternative of in-feed colistin sulfate to improve growth performance and intestinal microbial environment in newly weaned pigs. Anim Sci J.

[20] Cera KR, Mahan DC, Reinhart GA (1989). Postweaning swine performance and serum profile responses to supplemental medium-chain free fatty acids and tallow. J Anim Sci.

[21] Matsui H, Imai T, Kondo M, Ban-Tokuda T, Yamada Y (2021). Effects of the supplementation of a calcium soap containing medium-chain fatty acids on the fecal microbiota of pigs, lactating cows, and calves. Anim Sci J.

[22] Park S, Sun S, Wongchanla S, Chen Y, Li X, Liu Y (2025). Dietary supplementation of blend of organic acids and monoglycerides alleviated diarrhea and systemic inflammation of weaned pigs experimentally infected with enterotoxigenic Escherichia coli F18. J Anim Sci Biotechnol.

[23] Lee SI, Kang KS (2017). Function of capric acid in cyclophosphamide-induced intestinal inflammation, oxidative stress, and barrier function in pigs. Sci Rep.

[24] Miyagawa Y, Mori T, Goto K (2018). Intake of medium-chain fatty acids induces myocardial oxidative stress and atrophy. Lipids Health Dis.

[25] Świątkiewicz M, Hanczakowska E, Okoń K, Kowalczyk P, Grela ER (2020). Effect of maternal diet and medium chain fatty acids supplementation for piglets on their digestive tract development, structure, and chyme acidity as well as performance and health status. Animals.

[26] Funderburke DW, Seerley RW (1990). The effects of postweaning stressors on pig weight change, blood, liver and digestive tract characteristics. J Anim Sci.

